# The Cognitive and Emotional Aspect in Fibromyalgia: The Importance of the Orofacial Sphere

**DOI:** 10.7759/cureus.36380

**Published:** 2023-03-19

**Authors:** Bruno Bordoni, Allan R Escher, Gianmarco Cannadoro, Filippo Tobbi

**Affiliations:** 1 Physical Medicine and Rehabilitation, Foundation Don Carlo Gnocchi, Milan, ITA; 2 Anesthesiology/Pain Medicine, H. Lee Moffitt Cancer Center and Research Institute, Tampa, USA; 3 Cardiology, Don Carlo Gnocchi Onlus, Milano, ITA; 4 Osteopathy, Poliambulatorio Medico e Odontoiatrico, Varese, ITA

**Keywords:** inflammation, osteopathic, temporomandibular joint, dental occlusion, pain, fibromyalgia

## Abstract

Fibromyalgia syndrome (FMS) is a systemic and multifactorial disease of unknown etiology. There are many co-morbidities that the patient presents, making the clinical picture not immediate. Cognitive decline and emotional alteration (anxiety and depression) are found in fibromyalgic patients, as well as temporomandibular joint arthrokinematic disorders, dental malocclusion, and periodontitis. There seems to be a biunivocal relationship between oral and psychiatric dysfunctions in fibromyalgia. The article reviews the information regarding oral health alterations with respect to the patient's cognitive and emotional response, as the most recent information we have raises new hypotheses about the diagnosis of FMS.

## Introduction and background

Fibromyalgia syndrome (FMS) is a systemic disorder, affecting approximately 0.5-6.6% of the world population [1,2]. The syndrome is characterized by multiple symptoms, widespread chronic pain, fatigue, not restorative sleep, emotional and cognitive alterations, balance disorders, muscle-joint stiffness, kinesiophobia, and vertigo [1-3]. Fibromyalgia is classified as a rheumatological disorder of unknown etiology and is indicated as the second most common pathology compared to osteoarthritis [4].

The etiology of FMS is multifactorial, with evidence of subgroups of patients with genetic alterations, dysfunctions of hormonal and metabolic systems, and systemic and local neurological disorders [5]. Symptoms negatively impact patients' quality of life and social and work relationships [6]. For a correct diagnosis of FMS, two years can pass from the onset of symptoms, with a tendency to involve the female sex in this pathology with unclear boundaries [3]. Many overlapping symptoms, such as the concomitant occurrence of irritable bowel syndrome, interstitial cystitis, orthostatic intolerance, dry mouth, and dry eyes, Reynaud's syndrome, celiac disease, obesity, sexual dysfunction, glucose intolerance and diabetes, oral area dysfunction (pain and altered arthrokinetics of the temporomandibular joint, malocclusion), intestinal dysbiosis and Crohn's disease [7-9].

There are various scales of evaluation and clinical classification for the patient with FMS; Revised Fibromyalgia Impact Questionnaire (FIQR), Fibromyalgia Assessment Status (FAS), Polysymptomatic Distress Scale (PDS), Nociplastic-based Fibromyalgia Features (NFF), Symptom Severity Score (SSS), Widespread Pain Index (WPI) [9,10]. FMS is not a diagnosis of exclusion, and according to the 2016 American College of Rheumatology guidelines, a diagnosis can be made if the patient has a WPI of ­≥7 and an SSS with ≥5, or, WPI 4-6 and a SSS ≥9; if the patient shows generalized pain involving at least 11 body areas (18 areas in total), and with symptoms present for at least 3 months [11]. FMS is characterized by an alteration of pain-processing pathways, peripherally and centrally. Central sensitization leads to phenomena such as allodynia and hyperalgesia; there is an increase in excitatory neurotransmitters (substance P and glutamate), with a parallel decrease in the levels of medullary substances of the descending and ascending anti-nociceptive pathways (serotonin and norepinephrine, endogenous opioids) [12].

Some brain areas that handle pain information are more active than in healthy subjects, such as the posterior insula, secondary somatosensory cortex [12]. Not only is an increased activity found, but the same areas may experience less connectivity with brain areas that filter nociceptive afferents more, producing an ineffective response in reducing antinociceptive afferents to the spinal cord [12]. Three types of pain are recognized in rheumatology. Nociceptive pain, which results from an injury to a tissue (joints), is a type of sensation typical of rheumatoid arthritis. Neuropathic pain results from a lesion of nerve tissue as in Sjögren's syndrome and psoriatic arthritis. FMS pain is of the nociplastic type, i.e., an alteration in the processing of non-painful sensations transformed into pain (allodynia, hypersensitivity). The article reviews the information regarding oral health alterations with respect to the patient's cognitive and emotional response, as the most recent information we have raises new hypotheses about the diagnosis of FMS.

## Review

Orofacial sphere dysfunction and fibromyalgia

One of the dysfunctions that the patient with FMS reports is an alteration of the function of the temporomandibular joint (locking, clicking), with a greater prevalence in the female sex [13]. A recent observational study with 300 patients, of which 150 were diagnosed with fibromyalgia, highlighted that patients suffer more from bruxism and teeth grinding than healthy subjects [13]. The dysfunction is linked to the presence of FMS, with increased muscle pain in the contractile districts related to chewing and within the joint [13,14]. Other symptoms related to the orofacial area are xerostomia, dysgeusia, and glossodynia. Xerostomia involves a high percentage of patients, with a maximum of 71% of findings; this condition can favor the onset of infections of the teeth and gums and create the conditions for an altered dental occlusion [15]. Malocclusion is defined as an alteration of the relationship between the upper and lower first molars; this condition lays the foundations for the detection of oral infections, becoming a vicious circle [16]. The same infections can alter the arrangement and presence of the teeth, causing malocclusion [17]. In the literature we do not have extensive data on oral dysfunctions in fibromyalgia patients, despite the important consequences that can arise and the high percentages of findings (42%-94%) [13,18-20]. Can oral area disorders be one of the causes that lead to the development of FMS? Can oral dysfunctions arise before the diagnosis of FMS? These are different clinical questions to get a different view that could help the doctor and other health professionals to better perceive the presence or absence of fibromyalgia.

The relationship between orofacial disorders and fibromyalgia: periodontitis

A clinical feature of FMS is the presence of psychiatric disorders (depression, anxiety, cognitive impairment, panic), with a three times higher incidence than in the general population [15]. Patients with fibromyalgia have a substance P level three times higher than healthy subjects; substance P modulates the intervention of the N-methyl-d-aspartate receptor (NMDAR) at the thalamic level, stimulating thalamic mast cells [15,21]. NMDAR, together with other neuromodulators (hemokinin-1), and pro-inflammatory substances (cytokine 6 and tumor necrosis factor-alpha), stimulates the thalamic production of neurosensitizing molecules (histamine, interleukin 1beta and 6, tumor necrosis factor-alpha, calcitonin- gene related peptide, hemokinin-1, substance P) [21]. This metabolic environment directly stimulates the pain receptors of the thalamus, or indirectly, via the glial cells of the diencephalon, creating a situation of central sensitization with systemic inflammation and pain (neuroinflammation) [15,21]. Substance P is one of the molecules involved in depression and anxiety, and in cognition [22,23].

Neuroinflammation causes cognitive impairment and psychiatric disorders [24]. Chronic local and systemic inflammation, as well as chronic infections, predispose the person to develop rheumatic diseases, including fibromyalgia [25]. Periodontitis is a local and systemic infection/inflammation. There is a relationship between periodontitis and the onset of FMS, and between fibromyalgia patients and the detection of periodontitis: the relationship is biunivocal [25]. During the development of periodontitis, substance P (and neurokinin A) stimulate mast cells locally and then systemically, elevating inflammatory parameters [25]. The presence of periodontitis should be a marker or red flag during a screening for evaluation of fibromyalgia, as it could be a symptom or one of the causes leading to FMS [25].

**Figure 1:**
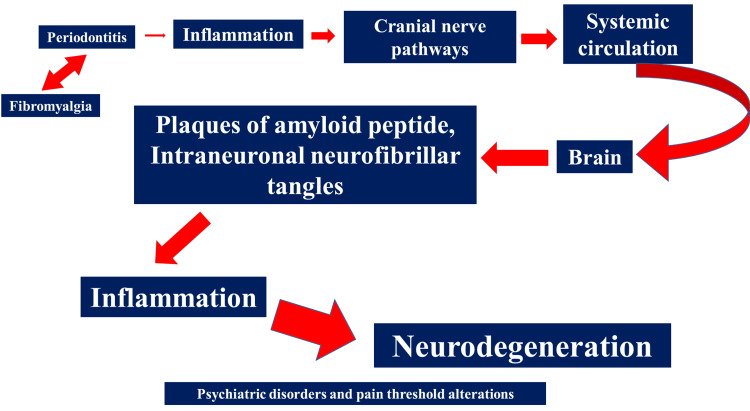
A schematic of how periodontitis can cause neurodegeneration, resulting in cognitive decline and psychiatric disorders.

The same American College of Rheumatology describes how the presence of temporomandibular joint alterations and dry mouth are clinical indications of the presence of FMS [25]. Infections/inflammations of the gingival tissue are thought to cause a systemic disturbance to the central nervous system, introducing bacterial and viral substances into the brain area (via circulation and cranial nerve pathways), which in cascade stimulate inflammatory metabolic reactions [26]. Periodontitis affects half of the population and can cause increases in mortality and morbidity (cardiovascular disease, diabetes); this orofacial disorder can cause a cognitive decline, probably due to chronic inflammatory reactions, which would cause the formation of intraneuronal neurofibrillar tangles and plaques of amyloid peptide [27]. There is a relationship between periodontitis, fibromyalgia, and psychiatric disorders (Figure 1).

The relationship between orofacial disorders and fibromyalgia: dental malocclusion

A dysfunction of the temporomandibular joint (TMJ), and/or the presence of periodontitis can cause alterations of dental occlusion, i.e., a discrepancy in the relationship between the teeth of the upper and lower arches [28,29]. The same mandibular joint pain can negatively affect the occlusion, which condition of impaired joint use involves just under 19% of patients with FMS [30]. The main afferents from the TMJ project to the trigeminal subnuclei interpolaris/caudalis transition zones (Vi/Vc), which is an important arrival station of nociceptive information, which starts to manage nociception [30,31]. The same nociceptive afferents send information to the ventromedial rostral Vi/Vc area of the spinal cord (C1-2) [30,31]. In an animal model, a malocclusion generates a lowering of the pain threshold, widening the hypersensitivity of somatic pain, typical of FMS [30]. A nociceptive increase causes an increase in cholecystokinin (CKK), a neuropeptide found in the central nervous system, which increases and stimulates the descending pain pathways from the periaqueductal gray (PAG) midbrain and ventromedial Vi/Vc-rostral spinal cord [30]. The increase of CCK in the medulla stimulates a cascade of interleukin-18 from glial cells, producing an additional inflammatory environment (elevation of tumor necrosis factor-alpha, interleukin-1 beta levels) and pain [30]. These mechanisms create a central sensitization, starting from malocclusion, on an animal model, favoring the activation of descending pain pathways [30].

A masticatory dysfunction can induce behavioral and cognitive alterations. In an animal model, a malocclusion generates a morpho-functional remodeling of the hippocampus area, reducing memory and learning ability, inducing anxiety and depression; in the human model, an alteration of dental occlusion lays the foundations for dementia [32]. The information sent by the stimulation of the periodontal ligaments (proprioceptive information) on a basis of altered occlusion (via trigeminal pathways), will stimulate the trigeminal mesencephalic nucleus (Vme), which will send afferents towards the ventral postero-medial area of the thalamus (VPM); the latter will send information to the primary somatosensory area (S1), a fundamental center for the management of emotions (received and emitted) and with a close relationship with the limbic area [32]. The afferents thus created (malocclusion) will return as non-physiological afferents, altering behavior (anxiety and depression).

There is a strong connection between proprioception and emotions; not only does a dysfunctional somatic structure generate pain, but also non-physiological emotions. Additionally, when systemic neuroinflammation exists (as in fibromyalgia), area S1 is overstimulated [32]. Another pathway that could generate anxiety and depression from the presence of malocclusion is the stimulation of the habenula. The habenula lies above the posterior terminal portion of the thalamus [33]. The lateral area of the habenula (LHb) is involved in emotion modulation [33]. Vme sends afferents to LHb, whereas, in the presence of malocclusion, the constant excitation of LHb translates further afferents towards Vme; this circuit facilitates the intervention of the trigeminal motor nuclei, creating a vicious circle between the hyper-excitation of the masticatory muscles on the basis of malocclusion and anxiety and stress, fueling behavioral disorders [33].

**Figure 2:**
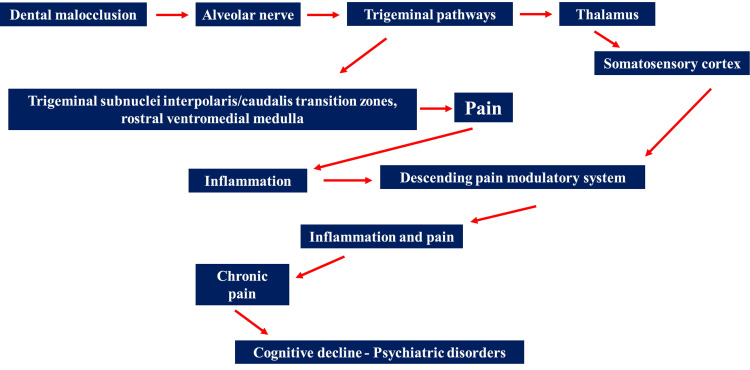
Schematic illustration of the relationship between the presence of dental malocclusion and the local and systemic responses that a chronic non-physiological dental contact causes in the patient. These neurological and metabolic links could explain the persistence of certain pathological patterns in the fibromyalgia patient or pose new hypotheses on the onset of fibromyalgia. This scheme reflects the content of the text.

Another hypothesis is to understand how a non-physiological proprioceptive stimulation can induce emotional dysfunctions. For example, impaired proprioception is probably an important stimulus for creating or fueling morpho-functional changes in the central and peripheral nervous system, leading to or increasing inflammation and pain, cognitive decline, and behavioral disturbances. In patients with FMS the peripheral proprioceptive system appears dysfunctional (Figure 2) [6].

We recall that the presence of depression and anxiety in patients with FMS is correlated with cognitive impairment [34]. Furthermore, there is a close relationship between a dysfunction of the (little inhibited) descending pathways that modulate pain perception and cognitive decline [35]. This lack of inhibition is caused, among other causes, by the elevation of brain-derived neurotrophic factor (stimulated by neuroinflammation) in the periaqueductal gray area, stimulating the tropomyosin receptor kinase B (TrkB) receptors of the rostral ventromedial medulla; this mechanism allows the excitability of the descending pain pathways to persist [35].

There are many questions still unanswered. To give examples, we do not know the real connections between a malocclusion and the concomitant presence of systemic pathologies (heart disease, rheumatism, respiratory disease, diabetes, malignant tumors, rhinitis, otitis) [36]. In an animal model, a malocclusion stimulates an altered pancreatic response, with non-physiological glucose values. Probably, the malocclusion sends aberrant afferents to the midbrain trigeminal nucleus and the dorsal motor nucleus of the vagus nerve; the returning efferents will influence neuroendocrine responses that may pave the way for diabetes [37].

To conclude with examples, malocclusion stimulates afferents to the sensory trigeminal nucleus (animal model), which projects information to the cerebellum, influencing posture and balance [38]. The diagnostic approach and treatment of fibromyalgia should always be multidisciplinary, as the areas involved in FMS do not concern only the musculoskeletal area. Future research should make further efforts to improve the clinical picture of the fibromyalgia patient, also taking into account the information that the literature presents in the field of oral dysfunctions. These dysfunctions could, in fact, not only cause the persistence of the symptoms but also determine their onset.

## Conclusions

Fibromyalgia syndrome (FMS) is a systemic disorder, with symptoms not only somatic (muscular and joint) but also within the cognitive and psychiatric spheres (anxiety and depression). The correct clinical picture is not immediate and, on some occasions, the diagnosis of FMS can occur after a few years. The article reviewed some information regarding orofacial system dysfunctions, such as periodontitis and dental impaction, related to fibromyalgia. Alterations of the orofacial sphere could be not only the cause of the symptomatological persistence of the fibromyalgic patient but also a cause that would stimulate the onset of FMS. During the patient's visit, the history of the orofacial area should not be missing and should not exclude in advance that the presence of disorders such as periodontitis and malocclusion could be signals to better address the diagnosis. To conclude, dentists should collaborate with rheumatologists if the patient presents multiple symptoms, with the ultimate aim of preventing or better directing the therapeutic procedure. Further effort should be made by the clinician to broaden the knowledge of this pathology.
